# Level of distress, somatisation and beliefs on health-disease in newly arrived immigrant patients attended in primary care centres in Catalonia and definition of professional competences for their most effective management: PROMISE Project

**DOI:** 10.1186/1471-2296-14-54

**Published:** 2013-05-04

**Authors:** Pere Torán-Monserrat, Jordi Cebrià-Andreu, Josep Arnau-Figueras, Jordi Segura-Bernal, Anna Ibars-Verdaguer, Josep Massons-Cirera, Mª Carmen Barreiro-Montaña, Sandra Santamaria-Bayes, Esther Limón-Ramírez, Juan José Montero-Alia, Carles Pérez-Testor, Guillem Pera-Blanco, Laura Muñoz-Ortiz, Carolina Palma-Sevillano, Gerard Segarra-Gutiérrez, Sergi Corbella-Santomà

**Affiliations:** 1 Primary Healthcare Research Support Unit Metropolitana Nord, IDIAP Jordi Gol, Carrer Major 49-53, 08921 Santa Coloma de Gramenet, Spain; 2 Primary Healthcare Centre Gatassa (Mataró 6), Catalan Health Institute, Camí del Mig 36 (2ª planta), 08303 Mataró, Spain; 3 Department of Medical Sciences, Girona University, Carrer Emili Grahit 77, 17071 Girona, Spain; 4 Department of Education, Generalitat de Catalunya, Territorial Services in Vallès Occidental, Carrer Marqués de Comillas 67-69, 08202 Sabadell, Spain; 5 Blanquerna Faculty of Psychology, Education Sciences and Sport, Ramon Llull University, Carrer Císter, 34, 08022 Barcelona, Spain; 6 Primary Healthcare Centre Ronda Prim (Mataró 7), Catalan Health Institute, Camí del Mig 36 (3ª planta), 08303Mataró, Spain; 7 Primary Healthcare Centre Rocafonda Palau (Mataró 3), Catalan Health Institute, Ronda Pintor Rafael Estrany 24, 08304 Mataró, Spain; 8 Mental Health Vidal i Barraquer Institute, Ramon Llull University, Carrer Sant Gervasi de Cassoles 88, 08022 Barcelona, Spain

## Abstract

**Background:**

Newly arrived immigrant patients who frequently use primary health care resources have difficulties in verbal communication. Also, they have a system of beliefs related to health and disease that makes difficult for health care professionals to comprehend their reasons for consultation, especially when consulting for somatic manifestations. Consequently, this is an important barrier to achieve optimum care to these groups. The current project has two main objectives: 1. To define the different stressors, the level of distress perceived, and its impact in terms of discomfort and somatisation affecting the main communities of immigrants in our area, and 2. To identify the characteristics of cross-cultural competence of primary health care professionals to best approach these reasons for consultation.

**Methods/Design:**

It will be a transversal, observational, multicentre, qualitative-quantitative study in a sample of 980 people from the five main non-European Union immigrant communities residing in Catalonia: Maghrebis, Sub-Saharans, Andean South Americans, Hindustanis, and Chinese. Sociodemographic data, level of distress, information on the different stressors and their somatic manifestations will be collected in specific questionnaires. Through a semi-structured interview and qualitative methodology, it will be studied the relation between somatic manifestations and particular beliefs of each group and how these are associated with the processes of disease and seeking for care. A qualitative methodology based on individual interviews centred on critical incidents, focal groups and *in situ* questionnaires will be used to study the cross-cultural competences of the professionals.

**Discussion:**

It is expected a high level of chronic stress associated with the level of somatisations in the different non-European Union immigrant communities. The results will provide better knowledge of these populations and will improve the comprehension and the efficacy of the health care providers in prevention, communication, care management and management of resources.

## Background

Family medicine and paediatric health care centres in Catalonia attend an important number of new-arrivals. It has been calculated that more than one million immigrants who have arrived in the last ten years have, at present, steady settlement [1]. From different countries, with different languages, cultures and backgrounds (often very different) there are many differences on how they explain the causes of their diseases [2,3].

The profile of immigrants in our setting is well known. Generally, they are young individuals from developing countries [4,5] who have already suffered psychosocial distress in their birthplace. They had to emigrate in precarious conditions and bearing great sacrifices, not only for themselves but also for their families and the community to which, in some way, they remain indebted. All this must be added the challenge to adapt to the new hosting country. Thus, initially, they have in common high level of distress [6-8].

Distress may be defined as the stress felt as unpleasant, normally due to the perception that demands to which the individual is submitted in specific situations widely exceed their resources to confront them [9]. Many convergent factors are involved for most immigrants. Newly arrived migrants face difficult experiences during settlement as the difficult conditions found in the host country, are different from what they expected and often disappointing. These issues are exacerbated by intense chronic stress that leads to serious psychological difficulties to face the process of adaptation to the new situation [10,11].

During the course of resettlement, numerous problems appear such as isolation, separation, lack of integration, cultural change, discrimination, currently unemployment and lack of minimum income [12]. This situation has a direct repercussion on their health status. In addition, it should be considered as influential to their health status the poor housing conditions, overcrowding, nutritional status, the absence of family support, the climate, different food and toxic habits [13]. This, results in anxiety and depression that leads to somatisation.

These somatisations are presented with intense signs and symptoms, not explained by physical disease but by psychosocial distress. Studies carried out in different settings coincide in that around 50% of the immigrants suffer or will suffer from these problems [14-16]. This has also been found in our setting in the studies undertaken in Catalonia about the reasons of consultation [2,17,18]. Thus, the numerous consultations for sleep disorders, myalgia, cephalea, impotence or unspecific abdominal or chest pains are examples of symptoms of psychosocial suffering and are interpreted as the development of disease within their own cultural beliefs. In a percentage of cases this psychological discomfort leads to frank depression [19] and in others it may result in a state, defined by some authors, as demoralization [20]. Primary health care professionals face these somatisations in the consultation. Around one third of the general population consult for physical symptoms not presenting any organic disease [21]. No other professional has more comprehensive and less iatrogenic view of these phenomena. Nonetheless, this type of consultation is especially frustrating for both the professional and the patient. The family practitioner is permanently challenged to manage the uncertainty that any of these symptoms may actually correspond to the initial stage of a severe organic disease or that the patient is misusing the system. These situations are generators of professional stress. If the visits are repeated with a certain frequency by the same patient, the physician may start applying him the dangerous and iatrogenic label of “pest and annoying” [22].

On the other hand, patients and physicians usually are in deep disagreement about the causes, significance, severity and treatment of the symptoms [23], which, in the case of the immigrant population, may be further aggravated by idiomatic and cultural barriers. Therefore, the difficulty in the management of a patient with somatisations increases when the patient comes from another culture. Apart from the idiomatic barriers we must add the lack of cross cultural knowledge of service providers [24], which enormously complicates the understanding of the reason of consultation and the therapeutic approach to be undertaken. It Somatizations have a strong cultural component and are conditioned by elements such as religion, beliefs and myths about the origin and appearance of diseases, and values of each community [25].

The few studies on this subject in our setting indicate that the somatisation disorders are more prevalent among immigrant population and represent a large proportion of consultations. Paradoxically, this type of situation is under-evaluated in immigrant patients compared to the national population. In a study performed in an urban area it was observed that 32% of the native patients received some type of treatment for these disorders while this percentage was reduced to 19.8% for the immigrant patients [26].

The recent migratory flow of the non-EU population towards the European Union yields to increasingly more complex and diverse societies. Far from stopping, these migratory flows will remain constant in the coming years. Given the geopolitical situation of Spain it may be foreseen that a large fraction of this migratory pressure will arrive from Sub-Saharan Africa [27]. The management of health problems affecting this people represents a challenge to the health care system [28] and to the professionals who should develop their own cross cultural competences [29-31].

The main objective of the project is to attain better knowledge of the different stressors affecting the immigrant population, to quantify the level of distress of these individuals and to determine the patterns of somatic expression, establishing comparisons among the main migrant communities in Catalonia.

We also intend to determine the significance attributed to these expressions of psychological discomfort with the aim of improving the level of comprehension and efficacy of the health care providers with respect to both communication and the therapeutic management of these situations. On the other hand, to develop a map of the competences for the health care providers to attend these demands more efficiently [32,33].

## Methods/Design

### General hypothesis

Migration is associated with a continuous process of people’s adjustment to a series of changing situations. This continuous pressure on an adjustment effort generates overload and psychological suffering which may be expressed by somatic complaints with no organic basis. The level of chronic stress among the different communities of new arrival immigrants is expected to be high and it should correlate with the level of somatisation. The causes of these symptoms would vary from one ethnic group to another with differences in age and gender. The system of beliefs would be related to the type and level of somatisation and with the patterns of use of the health care system. The health care providers will define competences, to provide better management to these patients. This issue has not been addressed in their professional training.

### Specific hypotheses

• The psychosocial stressors of the immigrants are common to all studied groups regardless their origin.

• The level of distress and the somatisations co-vary positively.

• The peak of distress occurs around the second year of settlement in the hosting country.

• The attributions of disease are different in each ethnic group but the higher the level of education the more the results will tend to converge.

• The localization of the somatic symptoms differs by ethnic group but has a common denominator in each.

• Differences will be found in the distress perceived and in the types of somatisation according to the age and gender of each community studied.

• The different profiles of risk which may require greater socio-health care could be identified.

• Professional competences will be defined to perform a better evaluation and management of the somatisations, the stressors and the situations of stress in individuals who have recently migrated.

### General objectives

Phase I: To define the different stressors, the level of distress perceived, and its impact in terms of discomfort and somatisation affecting the main groups of immigrants in our area, with special attention to the attributions they make to their symptoms and the somatic correlates.

Phase II: To define the professional competences to provide better care to these processes to the new arrival immigrant population.

### Specific objectives

Specific quantitative objectives (Phase I - A):

1.- Evaluate the level of distress perceived in a sample of individuals from 5 immigrant communities: Maghrebis, Sub-Saharans, South Americans, Hindustanis and Chinese.

2.- Evaluate the self-perception of the stressor situations in their daily life.

3.- Determine the level and types of somatisation in each community according to age and gender.

4.- Identify the main risk profiles of having stress and disorders by somatisation among the new arrival immigrant population.

5.- Establish the differences between the group with somatisation disorder and non-somatisation (origin, level of distress, search for help).

Specific objectives of the qualitative study (Phase I – B):

1.- Determine the myths and symbolisms related to the symptoms and diseases of these groups.

2.- Knowing the health protective factors attributed in each community.

3.- Determine the immigrants’ mechanisms of seek for help to approach somatic symptoms and the associated distress in both the community and the health care system.

Specific objectives of the qualitative study to health professionals to define the competences (Phase II):

1.- Determine the factors of professional competence susceptible to improvement in the four axes:

• Cultural awareness and humility.

• Knowledge and cultural skills.

• Detection of clinical situations related to stress.

• Clinical management of the somatisations attended.

### Design

#### Phase I: Somatization and beliefs study

Transversal, multicentre qualitative and quantitative observational study, of the immigrant population residing in Catalonia.

##### Study setting

Primary healthcare centres at Maresme, Barcelonès and Vallès Oriental counties with a high percentage (around 20%) of immigrant population within their reference population.

##### Study subjects

Immigrants attended in the Primary Care Centres (PCC), over the age of 18 and less than 80 years of age, who have arrived to Europe within the last 10 years and belong to one of the 5 communities of greatest presence in the territory of the study setting:

• Maghrebis: Persons from Morocco, Tunis, Algeria.

• Sub-Saharans: Persons from Senegal, Gambia, Mali.

• South Americans: People from Bolivia, Ecuador, Colombia.

• Indo-Pak region: Persons from Pakistan, Afghanistan, Bangladesh

• Asians: Persons from China.

These communities represent more than 77% of the total population of immigrants in our region.

Inclusion criteria:

1. Male and female immigrants from 18 to 80 years of age from Morocco, Tunis, Algeria, Senegal, Gambia, Mali, Bolivia, Ecuador, Colombia, Pakistan, Afghanistan, Bangladesh and China.

2. Length of stay in Europe of a minimum of 6 months and a maximum of 10 years.

3. Having been attended in the Primary Care Centre at least once.

4. Accepting to participate in the study and providing informed consent.

Exclusion criteria:

1. Any difficulty in communication preventing the collection of reliable data even in the presence of a cultural mediator.

2. Sensorial disorders impeding the performance of the study questionnaires and interviews.

3. Not providing informed consent.

##### Sample size

Accepting an alpha risk of 0.05 for a precision of ± 7% and an estimated prevalence of immigrants with distress of 50% as the case of maximum indetermination, a sample size of 196 immigrants representative of each of the 5 study groups will be necessary. This represents a total of 980 individuals to be included by stratified, non proportional sampling by communities of immigrants. For the qualitative substudy at least 2 individuals from each strata formed by community, gender, age (< 35 years, ≥ 35 years), and time of residence in Europe (< 2 years, ≥ 2 years) will be selected, by convenience. Thus, at least 2 × 5 × 2 × 2 × 2 = 80 interviews or those necessary until all the information is satisfactorily obtained.

##### Study variables

Demographic data: origin (country and ethnic community), age, gender, educational level, religion, marital status, number of children, people in the household, length of stay in Europe (years of immigration), present occupation, employment situation (legal/illegal), grade of comprehension and use of the official languages of Catalonia (Catalan/Spanish).

For the quantitative study, dependent variables will be evaluated with the Spanish versions of the following scales:

– The adapted Demographic Psychosocial Inventory (DPSI) scale of sources of distress [34]. This scale has 30 items which explore the characteristics of daily life that may cause distress such as problems with housing, family or language, climate, etc. This scale has good parameters of internal consistence and a good correlation with the Brief Symptom Inventory (BSI).

– Brief Symptom Inventory [35]. This has 46 items related to a list of psychological symptoms through a Likert scale of 5 grades. It allows the detection of levels of distress perceived as well as psychological and somatic manifestations reactive to stressing situations. This scale has been translated and validated in Spanish and provides good parameters of reliability and validity.

– Somatic Symptom Inventory (SSI) [36,37]. This is a brief 26-item questionnaire which allows good discrimination between the forms of moderate and severe somatisation also allowing the detection of co-morbidity, hypochondria and anxiety.

The results of each of these scales will be a continuous quantitative variable and an ordinal categorical variable.

For the qualitative part of Phase I, a semi structured questionnaire will provide a free narrative book of the informants, their perceptions related to their most important preoccupations, disturbances and diseases, as well as on their system of beliefs linked to health and disease. This part of the interview will be taped and later transcribed for content analysis. The analysis of the narrative of the informants will be carried out based on the fundamentals of the Grounded Theory [38,39] related to qualitative methodology.

##### Pilot survey

A pilot survey will be previously undertaken in a reduced sample of the study population. These cases will be excluded from the main study to avoid data distortion. This study will allow the verification of the applicability of the tools to be used, to increase the skills of the interviewers and standardize the techniques to introduce and perform the interview. Thereby anticipating possible problems that may emerge during the field work, such as the time used in each interview or the problems of communication due to idiomatic barriers. The questionnaires will be totally homologous to those carried out in the main study and will be supported by the cultural mediators of the centres in which this pilot survey will be undertaken.

##### Data collection

Ten students in the last year of the Master of Psychology of Health will be recruited for the principal study. They will take a training course for field investigators specifically designed for the study and based on the information obtained in the pilot study. The aim of this 6-hour course will be to improve the competence of the interviewers especially in the qualitative part and ensure a standardized and homologous pattern of action during the interviews. The interviewers will be assigned to the Primary Care Centres involved in the study.

The cultural mediators/facilitators of each ethnic/national group will be contacted in each of the selected Primary Care Centres to ensure their availability for the field work.

Study individuals will be randomly recruited based on a list stratified by age, gender and origin. The interviews will be performed together with the cultural mediators of each community in a relaxed setting ensuring privacy and confidentiality.

##### Data analysis

Once the data have been cleaned, a descriptive analysis for each of the variables will be performed for the quantitative study, including: frequency and percentage for the categorical variables and means and standard deviation for the variables. A global mean score and standard deviation for each of the three scales (DPSI, BSI and SSI) will be given for each of the 5 communities. The three scales will also be treated as categorical variables. We will evaluate the bivariate relation between the score of each of the scales with the remaining variables defined using analysis of variance for the differences between means in quantitative and categorical variables and the χ^2^ test for comparisons of proportions in two categorical variables. Multiple linear regression will be carried out for the three scales with the aim of determining which of the variables studied are associated with higher scores (greater levels of distress and somatisation) taking the score returned by each of the scales as the dependent variable and the possible associated factors as mutually adjusted independent factors. When the scores of the scales are treated as categorical variables polytomic logistic regression will be used. An analysis of correlation will be undertaken between the different scales using the Pearson coefficient of correlation with the aim of determining a linear relation between the different scores of each scale. Statistical significance will be considered with a p < 0.05. Statistical analysis will be performed with the Stata/SE 11 version statistical programme (StataCorp, College Station, Tx, USA).

The qualitative data will be analysed twice using the analysis of content and the methodology of triangulation and expert judgement. Qualitative data will be transformed for quantitative analysis following the methodology of the Grounded Theory [38,39] aided by the Atlas Ti programme for qualitative analysis of text data.

#### Phase II: Professional competences study

The guidelines for the qualitative investigation of Phase II will be designed from the data obtained in Phase I. It will be focused on the professional competences necessary for correct care to immigrants facing different situations of distress. The axes directing this phase of investigation are:

1. The focus of interest is constituted by the significances and perceptions on what primary health care professionals do when they provide health care to an immigrant patient with a somatisation disorder.

2. The tasks to be performed are related to health care and are descriptive and interpretive.

3. Their character is inductive.

The following techniques will be used:

Personal interviews (PI), open, semi-structured and registered by tape, focused on critical incidents in daily practice. Forty health care providers will be selected (10 nurses, 10 primary care physicians, 5 primary care managers and 5 social workers) who work in Primary Care Centres and voluntarily participate in the study. They will be invited to participate in semi-structured personal interviews (PI) in which the professionals will be asked to identify their behaviour when providing care to immigrant patients with situations of distress. The estimated length of of the interviews is between 30 to 90 minutes. This method, introduced by Flanagan [40,41], registers incidents which, from the point of view of the person interviewed, are “very important” to describe the objective of the study. These incidents should be contextualized, that is, they should be related to the situation or problem to be approached. To do this a dossier with the results of the previous Phase I will be elaborated.

Interviews in Focus Groups (FG) and analysis of the content of the conversation. The participants of the FG will be called to a session led by one of the investigators and by a consultant who is an expert in the definition and evaluation of competences. It will last approximately 4 hours and will be taped, upon authorization of the participants. A research assistant will take notes and will compile the material generated during the session (blackboard, wallpaper, notes of the participants). The starting point for the FG will be the initial list of cross-cultural competences obtained after the analysis of the content of the PI.

*In situ* questionnaires (ISQ). This involves answering the questionnaires generated from the information produced through the two previous techniques in the in-depth personal interviews. The responses to these questionnaires will allow triangulation, of the information with the aim of correcting the terminology and reviewing the effect that the questionnaire may have on the informant. This technique will be conducted by one of the investigators after previously obtaining authorization from the participants and also will be taped and treated as in-group sessions.

##### Data analysis

A narrative analysis of the content and comprehensive material of the PI, FG and ISQ will be performed with the participation of the internal and external investigators. All the information generated will be transformed into digital format. The transcriptions of the personal and group interviews and the documents will be compiled into a single body. That includes collections of texts or images of oral discourses, notes and registries, reproductions of graphic elements and previously written texts or already existing documents. The interviews will be independently analysed by three members of the research team, respecting the expressions of the informers. The data will be segmented and encoded to be of potential relevance for the definition of the cross-cultural competences and care to immigrants in situations of distress. Analysis of this information will follow the proposal of the Grounded Theory [38,39].

### Ethics

The confidentiality of the data obtained will be absolute and scrupulous with no data which may identify the participants of the study questionnaires. The data will be filed in databases with totally anonymous information technology support and will be wholly analysed by persons other than the interviewers. The questionnaires will be heteroadministered with the participation of cultural mediators/facilitators and specially trained interviewers committed to maintaining the confidentiality of the information obtained in the study. The interviews will be carried out in an isolated room with a relaxed ambiance favouring the free and sincere narrative of each interviewee after first having obtained written informed consent. If necessary, cultural mediators/facilitators will be required to obtain such consent. Upon completion of the interview each participant will be asked if they wish to receive information of the results of their interview and, if the response is affirmative, a postal address will be requested with the only aim of sending a brief report of the results. The protocol of investigation has been reviewed and approved by the Ethical Committee and Clinical Investigation of the Primary Care Research Institute IDIAP Jordi Gol (Barcelona, Spain) on July 21th 2010.

## Discussion

This investigation project aims to increase the level of comprehension of immigration from a psychosocial perspective. Despite the growing migratory phenomenon in our country since many years ago, we continue to have insufficient information on these populations [42], their state of health, their beliefs that influence their health status and how primary health care providers should be more effective in the management of high level of distress.

The results of this investigation will increase the level of comprehension of the principal stressors affecting persons in their settlement process, the repercussions they have on their health status and their cultural beliefs to the signs and symptoms presented.

Considering that the total number of visits produced in primary health care by the immigrant population is already important, improvement on the knowledge of cultural schemes will allow better management of these situations saving time to the professionals and probably reduce the use of resources and useless complementary tests. The final objective is none other than to improve the quality of care and the efficiency of the system. The importance of providing special care to this first generation of newly arrived citizens in all possible psychosocial aspects should also be considered since has an impact on the social integration of second generations [43]. A good health care and social care system should take these factors into account [44]. On the other hand, the possibility of establishing profiles of greater risk by age, gender or origin may favour effective preventive management at both the health care level and the social care programmes.

The present study has a series of limitations, the main limitation being derived from the difficulty to access persons of an immigrant group, especially in some closed communities or in those with idiomatic or cultural barriers. This makes patient recruitment and the field works more difficult since they might be suspicious of the interviewees. To minimize this effect cultural mediators/facilitators from the community will collaborate, aiding the interviewers to explain the objectives and characteristics of the study and, if necessary, acting as interpreters/translators.

There is the risk that the study groups present different demographic characteristics and that the distribution with respect to the study variables will not be homogeneous, and that the wealth of the symbolic of the interviewees regarding their conceptions on health and disease will be too complex for comprehension in a single interview. The performance of a pilot study and posterior analysis of the data obtained will provide better knowledge of these possible sources of bias to thereby implement the necessary corrector mechanisms.

The investigators involved in this project are aware that the study individuals represent an especially vulnerable group. Thus, all available means will be implemented to ensure that the interviews are perfectly respectful within the framework of mutual agreement and trust. The use of cultural mediators/facilitators from the same origin would overcome this problem. The process of selection of the students of the Master of Clinical Psychology and subsequent training of the field interviewers will facilitate the access to the interviewees and the attainment of information with better standards of quality.

We have foreseen the following axes of intervention with direct applicability based on the results obtained from the intervention proposed in this protocol:

• Establishment of risk profiles based on the characteristics of the migratory population or group from the five communities studied. The results will provide tools for health care planning to facilitate the identification of target groups in which active surveillance is required to prevent these situations of psychic suffering.

• Elaboration of a map of risk. The combination of the results obtained of the demographic data and of the census data of the immigrants will allow the elaboration of a map of risk of psychosocial suffering, which is an important element in the planning of services and for the assignment of resources to each territory.

• Analysis of barriers and facilitators in care to immigrants. The identification of barriers in the health care relation beyond the idiomatic barrier, based on real experiences lived by the immigrants will provide the elements to carry out an analysis of barriers and facilitators to thereby render better health care and social care.

• Establishment of plans of improvement in the quality of care to the immigrant from the primary care centres. We understand that a large part of the results obtained may constitute part of the analysis of the situation to thereby elaborate a plan of improvement in the team attending important members of these groups.

• Improvement on the efficiency of care provided. The study will allow improvement in the comprehension and efficacy of the professionals in their approach to these problems and the final result will represent a saving in time of consultation, costly and unnecessary diagnostic tests and referrals to specialists.

• Improvement on professional competences. Elaboration of guidelines to advice health providers on the management of these difficult situations in the consultation. Advanced training based in educational packages. Community interventions oriented towards early detection and prevention of this psychosocial suffering.

We therefore believe that the results obtained in this investigation will provide valuable knowledge to improve the comprehension of a prevalent health problem in our setting and will improve the professional practice to care immigrant patients.

## Abbreviations

BSI: Brief Symptom Inventory; DPSI: Demographic Psychosocial Inventory; EU: European Union; FG: Focus Group; ISQ: In situ Questionnaire; PHC: Primary Healthcare Centre; PI: Personal Interview; PROMISE: Promotion in recent migrating populations of the best management of somatisations and evaluation project; SSI: Somatic Symptom Inventory.

## Competing interests

The authors declare that they have no competing interests.

## Authors’ contributions

PTM, JCA, JSB contributed to formulating the research question, conception and study design. PTM is the coordinator of the investigation and has participated in the elaboration of the protocol of investigation and the writing of this article. JCA has elaborated and written the protocol of the study and has coordinated the tasks of the different work subgroups as well as being responsible for reference updating. JCA also designed the pilot study and has aided in the writing of the present article. JAF is in charge of the design and logistics of the substudy on professional competences (Phase II). JSB has contributed to the design of the Project and is responsible for the logistics of the field work and the selection of the interviewers. JMC, MBM, ELR, and JJMA have provided ideas for the design of the study and the systematic inclusion of cases in the pilot study and thereafter in the study and have contributed in the reporting of the results. CPT provided knowledge and experience in the scales of measurement and assessment in the qualitative methodology. GP and LMO supervised the methodology of the protocol of investigation and will be responsible for the treatment of the data and statistical analysis. CPS, GSG and SCS will contribute to the writing of the manuscript and the methodology of the analyses of qualitative data obtained throughout the project. All the authors have read, revised and approved the final manuscript.

## Pre-publication history

The pre-publication history for this paper can be accessed here:

http://www.biomedcentral.com/1471-2296/14/54/prepub
